# Two Potential Syphilis Vaccine Candidates Inhibit Dissemination of *Treponema pallidum*

**DOI:** 10.3389/fimmu.2021.759474

**Published:** 2021-11-25

**Authors:** Man Xu, Yafeng Xie, Kang Zheng, Haodang Luo, Manyi Tan, Feijun Zhao, Tiebing Zeng, Yimou Wu

**Affiliations:** ^1^ Institution of Pathogenic Biology, Hengyang Medical School, University of South China, Hengyang, China; ^2^ Hunan Province Cooperative Innovation Center for Molecular Target New Drug Study, University of South China, Hengyang, China; ^3^ Department of Clinical Laboratory, The Second Affiliated Hospital, Hengyang Medical School, University of South China, Hengyang, China; ^4^ Department of Clinical Laboratory, Hengyang Central Hospital, Hengyang, China; ^5^ Department of Clinical Laboratory, The Affiliated Nanhua Hospital, Hengyang Medical School, University of South China, Hengyang, China; ^6^ Department of Toxicology, Hunan Provincial Center for Disease Control and Prevention, Changsha, China

**Keywords:** *Treponema pallidum*, Tp0136, Tp0663, vaccine candidate, dissemination

## Abstract

Syphilis, caused by the spirochete *Treponema pallidum* subspecies *pallidum*, continues to be a major public health problem worldwide. Recent increases in the number of syphilis cases, in addition to the lack of an efficient vaccine against *T. pallidum* for humans, highlights an urgent need for the design and development of an efficacious syphilis vaccine. Here, we assess the vaccine potential of the adhesion protein Tp0136 and the outer membrane protein Tp0663. Rabbits were subcutaneously immunized with recombinant proteins Tp0136, Tp0663, or control PBS. Immunization with Tp0136 or Tp0663 generated a strong humoral immune response with high titers of IgG, as assessed by ELISA. Moreover, animals immunized with Tp0136 or Tp0663 exhibited attenuated lesion development, increased cellular infiltration at the lesion sites, and inhibition of treponemal dissemination to distant organs compared to the unimmunized animals. These findings indicate that Tp0136 and Tp0663 are promising syphilis vaccine candidates. Furthermore, these results provide novel and important information for not only understanding the pathogenic mechanisms of spirochetes, but also the development of spirochete-specific subunit vaccines.

## Introduction

Syphilis, caused by the spirochetal bacterium *Treponema pallidum* subsp. *pallidum* (*T. pallidum*), continues to be a globally prevalent disease, with an estimated burden of 36 million cases worldwide (1). In recent years, the rate of infectious syphilis has been sharply rising among men who have sex with other men (2–7). The incidence of congenital syphilis infections is also on the rise, with an estimated 1.36 million pregnant women infected worldwide each year, and approximately 520,000 of these pregnancies result in adverse outcomes (8). Moreover, syphilis also increases the risk of HIV transmission and acquisition (9). Despite the continued sensitivity of *T. pallidum* to treatment with penicillin, there is an urgent need for the development of an efficacious syphilis vaccine to complement the traditional screening and treatment approaches for the global elimination of syphilis, especially considering the increasing prevalence of syphilis worldwide.

Tp0136, a putative *T. pallidum* outer surface lipoprotein, has been implicated in treponemal dissemination (10). Tp0136 has been demonstrated to promote the migration of HMEC-1 cells and fibroblasts, contributing to the mechanism of chancre self-healing in syphilis (11, 12). Furthermore, Tp0136 is located on the *T. pallidum* surface, and as such, facilitates direct interaction with the host environment (10). Since *T. pallidum* Tp0136 has been shown to mediate attachment to plasma Fibronectin (Fn) and human cellular Fn (13), an abundant and ubiquitous ECM protein important for interactions between cells and the surrounding matrix, both of which are in close proximity to the vascular endothelium, interaction of Tp0136 with these molecules may contribute to facilitating *T. pallidum* dissemination *via* the bloodstream. Tp0663, a putative outer membrane protein that has surface-exposed epitopes (14), reacts strongly with serum antibodies from syphilis patients and *T. pallidum*-infected rabbits (15, 16), implying that it may have the potential to be a candidate vaccine.

To date, the development of a syphilis vaccine has achieved varying degrees of success (17, 18). Complete protection against *T. pallidum* challenge has been demonstrated only by immunizing rabbits with γ-irradiated whole cell *T. pallidum* preparations, which is considered an impractical immunization regimen (19). Partial protection, based upon attenuated lesion development, has been achieved in rabbits immunized with Tp0136 (10), TprI (20), TprK (21), Tp92 (BamA) (22), 4D (23), Gpd (24), TprF (25), endoflagella (26), and FlaB3 (27). Recent investigations have demonstrated that the immunization of rabbits with the laminin-binding adhesion protein Tp0751 protected against the dissemination of *T. pallidum* following intradermal challenge (28). Although these immunization regimens are impractical for use in humans, these studies indicate the importance of *T. pallidum* surface antigens and adhesins in conferring protection and significantly promote the development of a syphilis vaccine.

Here, we evaluated the immunoprotective capacity of Tp0663 and Tp0136 in a rabbit model of experimental syphilis. We showed that rabbits immunized with Tp0663 or Tp0136 displayed attenuated lesion development and exhibited a reduced treponemal burden in multiple distant organs when compared with the unimmunized rabbits. These findings demonstrated that Tp0663 or Tp0136 immunization inhibits treponemal dissemination to distant organ sites. Our results provide evidence that Tp0663 and Tp0136 may represent promising vaccine candidates for syphilis.

## Materials and Methods

### *T. pallidum* Propagation

Propagation of *T*. *pallidum* (Nichols strain) was performed as previously described (29). The bacteria were harvested from the treponemal suspensions by low-speed centrifugation to remove rabbit testicular debris.

### Recombinant Protein Expression and Purification

The *Tp0136 and Tp0663* genes, with an N-terminal hexahistidine tag, were separately cloned into the expression vector pET28a. The purification of recombinant Tp0136 and Tp0663 proteins was performed as previously described (10, 30), and then treated with Detoxi-Gel™ Endotoxin Removing Gel. Limulus amebocyte lysate (Chinese Horseshoe Crab Reagent Manufactory, Ltd., Xiamen, China) was used to detect the endotoxin in the Purified proteins, which were found to be less than 0.03 endotoxin unit (EU)/mL.

### Immunization Procedure

A cohort of nine male specific-pathogen free New Zealand White rabbits (2.5 – 3.0 kg, 13 – 15 weeks of age; The Animal Department of University of South China, Hengyang, China) with negative VDRL and FTA-Abs serology were selected for the immunization experiments. Rabbits were randomly divided into three groups (PBS control group, Tp0136-immunized group, and Tp0663-immunized group), with three animals in each group. Each animal was injected three times at two week intervals (0, 2, and 4 weeks) with 150 μg of test antigen or PBS emulsified in a 1:1 mixture with complete Freund’s adjuvant system (complete adjuvant for the first challenge and incomplete for the subsequent challenge; Sigma-Aldrich, St. Louis, MO, USA) at each immunization. The immunizations were divided equally among four subcutaneous and two intramuscular injection sites. The antiserum was collected to estimate the serum antibody titers.

### Intradermal Challenge

Twenty-one days after the final immunization, rabbits were sedated and intradermally challenged at eight sites along their shaved backs with 0.8 mL of 1×10^7^ freshly isolated *T. pallidum* (Nichols strain). Challenge sites were examined daily and photographed every three days to document lesion development. Dark-field microscopic (Nikon Canada, Mississauga, ON, Canada) was used to evaluate the presence of motile spirochetes in the lesion aspirates 21 days post-challenge.

### Analysis of a Specific Antibody Response to Recombinant Proteins

To evaluate the specific antibody response, 1 mL of blood was collected from rabbit ear veins in each group (n = 3, 3, 3) at 0, 2, 4 and 6 weeks following the first immunization. The blood was then centrifuged in order to separate the serum. The specific antibody levels were determined by indirect ELISA. Immulon microtiter 96-well plates (Thermo Labsystems, Franklin, MA) were coated at 4°C overnight with 10 μg purified recombinant protein. Excess antigen was washed off with PBS, and 200 μL of blocking buffer was added. After removal of blocking buffer, 100 μL of anti-serum was applied to the wells, starting with a 1/1000 dilution and further diluting the serum two-fold for each row of the plate. After 2 hours of incubation at 37°C, the wells were washed three times with wash buffer. Horseradish peroxidase (HRP)-conjugated goat anti-rabbit IgG (1:5000) was subsequently added to the wells and incubated at 37°C for 1 hour. The plates were rinsed three times with wash buffer, and 100 μL of TMB peroxidase substrate was added per well and incubated at 37°C for 15 minutes. Then 100 μL of 1 M sulfuric acid was added to stop the color reaction. Each experiment was repeated three times, and the absorbance was measured at a wavelength of 450 nm (BioTek Instruments, Winooski, VT, USA).

### Detection of Rabbit Interferon γ

The IFN-γ concentration in the serum collected from the rabbit ear veins after the last immunization were evaluated according to the manufacturer’s instructions by using an IFN-γ ELISA kit (Cusabio Biotech Co., Ltd., Wuhan, China).

### Extraction of *T. pallidum* DNA From Tissues and qPCR

The genomic DNA (gDNA) was extracted from *T. pallidum*-challenged rabbit tissues, including lesion biopsies at the local infection site, as well as liver, blood, spleen, and testicles, by using a DNeasy Blood and Tissue Kit (Qiagen, Shanghai, China) according to the protocol recommended by the manufacturer. Three tissue samples from each rabbit organ were analyzed for reproducibility. The gDNA was used as the template in the qPCR mixture according to the manufacturer’s standard protocol for QuantiFast SYBR one-step qPCR (Qiagen, Shanghai, China) and performed using a LightCycle 96 apparatus (Roche, Basel, Switzerland). Quantification of *T. pallidum* gDNA was determined using primers for the endoflagellar sheath protein (*flaA*) gene of *T. pallidum* and the collagenase-1 precursor (*MMP-1*) gene of rabbits (**Table 1**). A standard curve was created for *flaA* using a 10-fold serial dilution range from 10^7^ to 10^1^ copies of *T. pallidum* gDNA with an efficiency of 99.2% and an R^2^ value of 0.990. A standard curve was created for *MMP-1* using a two-fold serial dilution from 100 to 0.0488 ng/mL of rabbit gDNA with an efficiency of 99.1% and an R^2^ value of 0.995. The original gDNA concentration of rabbit tissue used to create the standard curve was obtained from spectrophotometric measurements. PCR conditions for *flaA* and *MMP-1* were as follows: initial denaturation at 95°C for 7 minutes, followed by 40 cycles of 95°C for 10 seconds, 56°C for 20 seconds, and 72°C for 20 seconds, A melt-curve analysis was performed with the following program: 95°C for 10 seconds, 65°C for 260 seconds, and 97°C for 1 second. Each assay was performed in triplicate. In addition, each assay was run with a no-template control.

**Table 1:** Primer sequences used in qPCR.

Gene	Organism	Sequence (5’ to 3’)	Annealing Temp (°C)
*flaA*	*T. pallidum*	F: AACGCAAACGCAATGATAAA	56
		R: CCAGGAGTCGAACAGGAGATAC	
*MMP-1*	Rabbit	F: TTGCTTCTTCACACCAGAATGCTGT	56
		R: TTGCTTCTTCACACCAGAATGCTGT	

### Histopathology

Tissues taken from skin lesions and testicles of each rabbit were fixed in formalin and embedded in paraffin, The tissues were then stained with H&E to analyze the abundance of immune cells, including lymphocytes, macrophages, and plasma cells, *via* microscopy.

### Statistical Analysis

Results are reported as means ± SD. All comparisons of differences between the test and control groups were assessed *via* the Students *t* test using GraphPad Prism 6.0 software (GraphPad Software, Inc., La Jolla, CA). *P* < 0.05 indicated a significant difference.

## Results

### Antibody Response of Rabbits to Immunization With Recombinant *T. pallidum* Proteins

**Figure 1:**
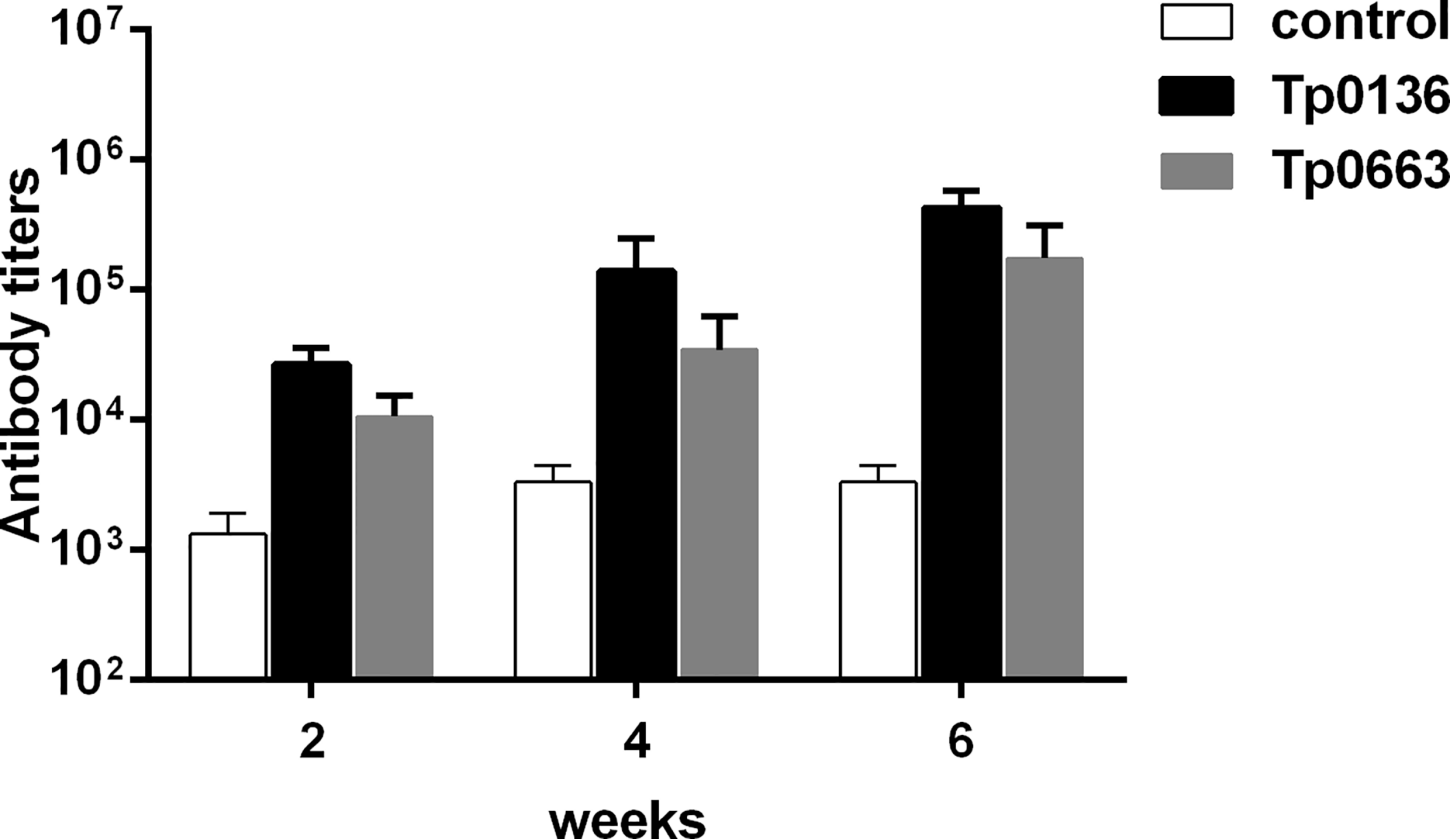
Antibody titers in rabbits immunized with recombinant Tp0136 or Tp0663 protein. New Zealand rabbits were immunized with 150 μg of recombinant protein Tp0136 or Tp0663 emulsified in complete Freund’s adjuvant system. The control rabbits were immunized with PBS emulsified in complete Freund’s adjuvant system. Each animal was injected three times at two week intervals (0, 2, and 4 weeks). Blood was drawn from the rabbit ear veins at 2, 4, and 6 week, and the IgG antibody levels were determined by indirect ELISA. Each set of data is based on measurements derived from three rabbits. To determine antibody concentrations (titers), two-fold serial dilutions of serum were made and the endpoint titer was considered to be the last serum dilution with readings higher than the mean + 3SD of the negative controls.

To determine whether *T. pallidum* membrane proteins Tp0136 and Tp0663 were able to induce specific antibody response in rabbits, we performed serial two-fold dilutions of serum to detect specific antibody titers. As shown in **Figure 1**, Tp0136 and Tp0663 induced high titers of anti-Tp0136 and anti-Tp0663 antibodies starting at week 2 compared with PBS-immunized rabbits. Moreover, antibody levels after the first and second immunizations were higher in animals immunized with Tp0136 than in animals immunized with Tp0663. These results indicate that *T. pallidum* membrane proteins Tp0136 and Tp0663 are able to induce specific antibodies.

### Tp0136 or Tp0663 Immunization Induced IFN-γ Secretion

**Figure 2:**
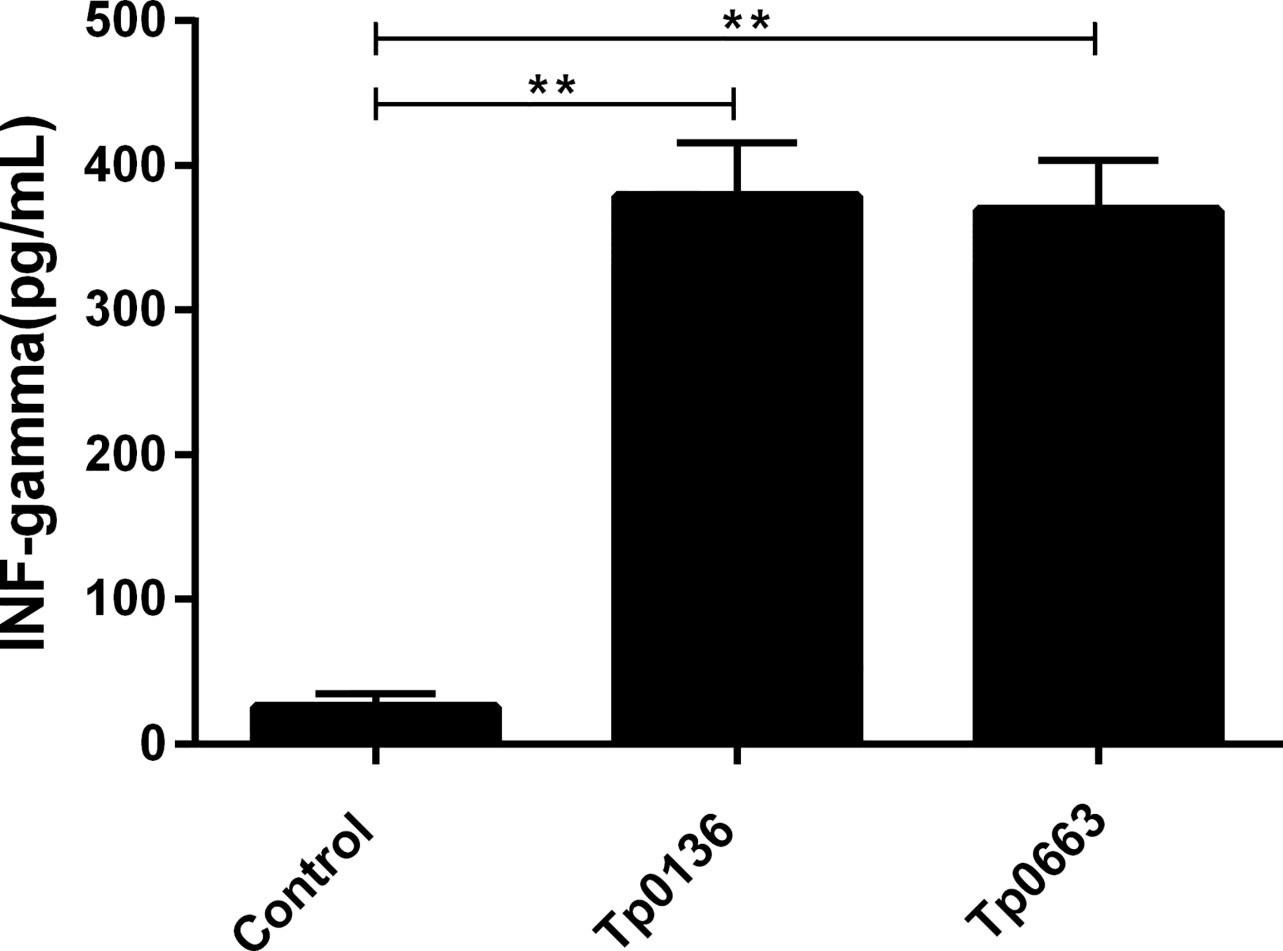
Production of IFN-γ in rabbits immunized with recombinant Tp0136 or Tp0663 protein. Following the final immunization, blood was collected from each rabbits’ ear vein. The IFN-γ level in the blood was evaluated by ELISA according to the manufacturer’s instructions. The results are expressed as the mean ± SD from three individual rabbits in each group. Each experiment was performed in three independent experiments (**P < 0.01).

Since Th1-related cytokines are critical to the efficient clearance of *T. pallidum* from lesion sites (31), it is important to assess the immunoprotective effect of a potential syphilis vaccine. In this study, serum was collected from Tp0136- or Tp0663-immunized rabbits following the final immunization. The levels of IFN-γ were then measured by ELISA (**Figure 2**). Compared to that of rabbits immunized with PBS alone, the production of IFN-γ was significantly increased in the rabbits immunized with Tp0136 or Tp0663.

### Tp0136 or Tp0663 Immunization Attenuated Lesion Development

**Figure 3:**
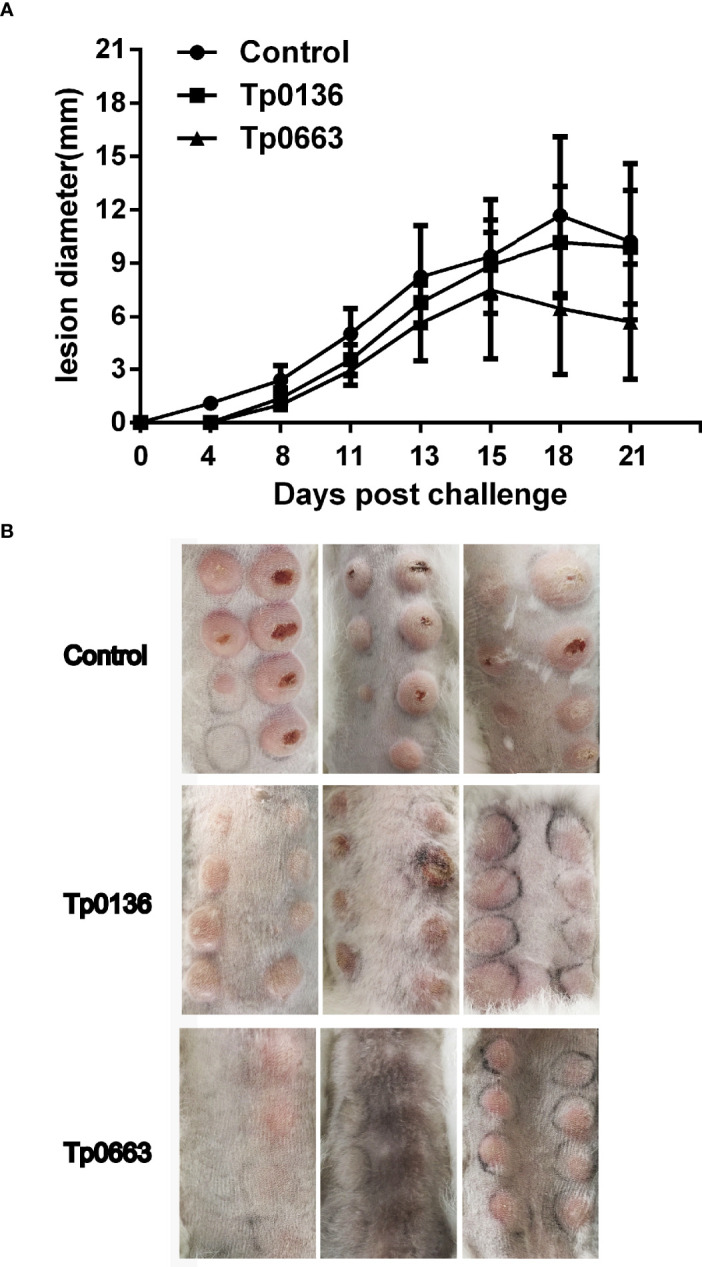
Immunization with recombinant Tp0136 or Tp0663 protein attenuated lesion development. **(A)** Lesion diameters were monitored over 21 days in the control, Tp0136-immunized or Tp0663-immunized animals following *T. pallidum* challenge. **(B)** Indurated lesions from eight locations on the backs of the rabbits were taken 21 days post-intradermal challenge with *T. pallidum* (Nichols strain).

Three weeks after the final immunization, all of the rabbits were sedated and intradermally challenged at eight sites along their shaved back with 0.8 × 10^7^ of freshly isolated *T. pallidum* (total of 0.8× 10^7^ *T. pallidum*/rabbit). During the subsequent 21 days, the appearance of lesions at the site of challenge were monitored, since chancre are unique to syphilis and represent the first clinical sign of infection (32). Lesions were monitored every three days for diameter and ulceration. Obvious lesions were present on all of the rabbits by day 11; however, Tp0136- or Tp0663-immunized animals presented delayed lesion development and smaller-size swellings compared to the control group (**Figure 3A**). Moreover, 21 lesion indurations (total of 24 sites) presented on control animals on day 4-6, and 24 lesion indurations (total of 24 sites) presented on Tp0136-immunized animals by day 8. However, only 16 lesion indurations (total of 24 sites) presented on Tp0663-immunized animals by day 8 (**Table 2**). The lesion diameter peak observed in control animals and Tp0136 immunized animals appeared at day 18, while in the Tp0663-immunized group the lesion diameter peak appeared at day 15 (**Figure 3A** and **Table 2**). The lesion diameter measured in the control group was larger than that in the vaccine groups, especially in the Tp0663-immunized group over the 21-day measurement period (**Figure 3A** and **Table 2**). Ulceration in the control group was apparent, and 85.7% of the lesions were ulcerated on day 21 (**Figure 3B** and **Table 2**). However, animals immunized with Tp0136 or Tp0663 presented only three ulcerations or no ulcerations, respectively, as compared to the control animals on day 21 (**Figure 3B** and **Table 2**). At day 21 post-challenge, dark-field microscopy was performed to evaluate the presence of viable *T. pallidum* in every lesion. As shown in **Table 2**, the ratio of lesions positive for motile *T. pallidum* in the control group was significantly higher than those in the two immunized groups.

**Table 2:** Lesion data for control and immunized animals.

Immunogen	Number of lesions/number of sites (%)*^a^*	Number of DF-positive lesions/total lesions (%)*^b^*	Number of Ulcerative lesions/total lesions (%)*^c^*	Lesion status	
				Median day*^d^*	Diameter (mm)*^e^*
Tp0136	24/24 (100)	8/24 (33.3)	3/24 (12.5)	11 (4-18)	10.17 ± 3.12
Tp0663	16/24 (66.7)	4/16 (25)	0/16 (0)	11.5 (8-15)	7.5 ± 3.92
Control	21/24 (87.5)	17/21 (81)	18/21 (85.7)	13 (8-18)	11.67 ± 4.42

**^a^** The denominator indicates the total number of challenge sites.

**^b^** The denominator indicates the total number of lesions examined by dark-field. microscopy. DF, dark-field.

**^c^** The denominator indicates the total number of lesions in the treatment group.

**^d^** The median day of lesion development with the range in parentheses.

**^e^** The maximal lesion size of lesions (Mean ± SD) in the treatment group.

### Tp0136 or Tp0663 Immunizations Inhibited *T. pallidum* Dissemination

**Figure 4:**
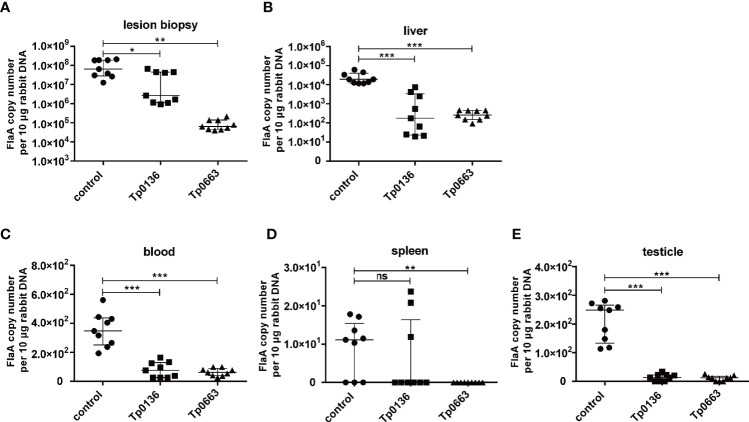
Immunization with Tp0136 or Tp0663 inhibited *T. pallidum* dissemination. Spirochete numbers were evaluated in control, Tp0136-immunized, or Tp0663-immunized animals by using qPCR to measure *flaA* DNA concentrations in lesion biopsies. The bacterial burdens in lesion biopsies are shown **(A)** at the local infection site, as well as in the disseminated infection sites of the **(B)** liver, **(C)** blood, **(D)** spleen, and **(E)** testicles at day 21 post-infection. Results were normalized within each tissue type based on the concentration of rabbit genomic DNA and presented as median ± interquartile range. Significance was assessed using Students *t* test (ns, not significant; *P* > 0.05,*P < 0.05, **P < 0.01, ***P < 0.001). Points correspond to three separately extracted tissue samples from each rabbit organ (nine total points in each group) were analyzed for reproducibility.

To determine whether Tp0136 or Tp0663 immunization protects against *T. pallidum* dissemination, all of the rabbits were killed at day 21 post-challenge. We then used real-time quantitative PCR (qPCR) to assess the burden of *T. pallidum* in biopsies from both primary and distal lesion sites, including blood, spleen, liver, and testicles. Analysis of the treponemal burden of the primary lesion sites revealed that *T. pallidum* DNA concentration in the three control animals was slightly increased compared with the Tp0136-immunized animals, but was substantially higher than Tp0663-immunized animals (**Figure 4A**). Moreover, we tested the treponemal burden in the blood of the rabbits, since *T. pallidum* is spread through blood. As shown in **Figure 4C**, we found that the treponemal burden in the blood was lower in the immunized animals than in the non-immunized controls. Owing to the propensity for *T. pallidum* to disseminate to distant organ sites, such as the spleen, liver, and testicles (33), we also analyzed the levels of *T. pallidum* in distant organ sites. Our results showed the capacity of *T. pallidum* to disseminate from primary lesion sites to distant organ sites (**Figures 4B, D, E**), with the liver and testicle extracts exhibiting a similar trend towards lower treponemal burden in all of the rabbits immunized with recombinant proteins. The most striking decrease was observed for the treponemal burden in the testicles of the vaccinated animals relative to the control animals (**Figure 4E**). Importantly, minimal treponemal DNA in spleen was detected in the Tp0663-immunized animals. Taken together, these results indicate that immunization with Tp0136 or Tp0663 inhibits *T. pallidum* dissemination to distant organ sites.

### Immunization With Tp0136 or Tp0663 Promoted Inflammatory Infiltration

**Figure 5:**
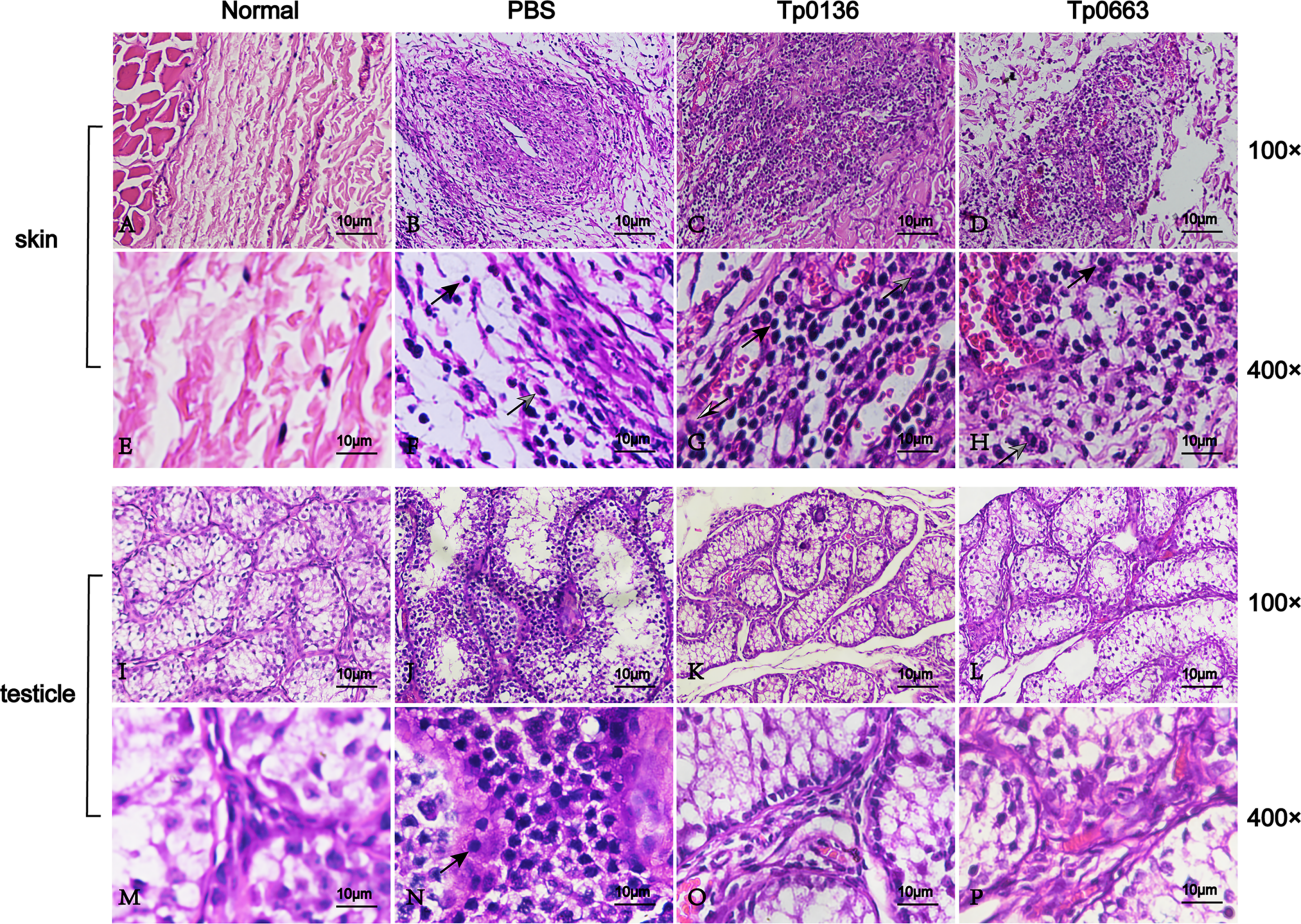
Histopathological changes of skin and testicle tissue after *T. pallidum* infection in rabbits. Skin and testicle tissues from normal tissue control **(A, E, I, M)**, PBS- **(B, F, J, N)**, Tp0136- **(C, G, K, O)**, or Tp0663-immunized rabbits (**D, H, L, P**, respectively) were sectioned and stained with H&E 21 days after infection with *T. pallidum*. Rabbits immunized with PBS and uninfected rabbits were used as controls. Inflammatory infiltrates in the skin and testicle tissue of rabbits were localized and predominantly lymphohistiocytic (black arrow = lymphocyte; black and white arrow = macrophage; gray arrow = neutrophil).

Since *T. pallidum* is thought to be cleared by macrophages *via* antibody-mediated opsonophagocytosis (34–36), hematoxylin and eosin (H&E) staining of biopsy samples obtained from incipient lesion sites of all of the animals was used to analyze the cellular infiltration, which was used to associate the decrease in the pathogen burden within primary lesion sites of vaccinated rabbits to immune cells (**Figures 5A**–**H**). Tp0136- or Tp0663-immunized rabbits had increased levels of immune cell infiltrate, including lymphocytes, macrophages, and plasma cells, relative to PBS-immunized rabbits (**Figures 5A**–**H**). Furthermore, analysis of the H&E-stained biopsy samples obtained from distant testicles revealed that inflammatory infiltrations were notably reduced in vaccinated animals compared to PBS-immunized animals (**Figures 5I**–**P**).

## Discussion

Although development of *T. pallidum* vaccines has achieved varying degrees of success (17) and long-term *in vitro* culture of *T. pallidum* has progressed greatly (37), no effective syphilis vaccine is currently available. One reason is that the molecular mechanisms underlying *T. pallidum* pathogenesis are poorly understood. Current research regarding the development of a syphilis vaccine focuses primarily on outer membrane proteins and adhesins. In this study, we investigated if immunization with the putative outer membrane protein Tp0663 and adhesion protein Tp0136 were able to induce a sufficiently strong and specific immune response in rabbits, thereby offering protection against *T. pallidum* infection. The results provide clear evidence that compared to unimmunized rabbits, rabbits immunized with Tp0136 or Tp0663 presented increased specific antibody titers, attenuated lesion development, increased cellular infiltration at the lesion sites, and inhibition of treponemal dissemination to distant organs.

During syphilis infection, primary lesions in human or in rabbits contain mostly macrophages, CD4^+^ T cells, CD8^+^ T cells, and natural killer (NK) cells (38, 39). A strong delayed-type hypersensitivity reaction, mediated by local type 1 (Th1) cytokines produced predominantly by CD4^+^ T cells, is critical to the efficient clearance of *T. pallidum* from primary lesions (40). The subsequent Th1 cytokine-mediated activation of resident macrophages promotes opsonophagocytosis of treponemes (31, 39, 41, 42), which is thought to be the major mechanism of clearance. The histological analysis of primary lesion sites in our study revealed an increase in the cellular infiltration in Tp0663-immunized and Tp0136-immunized animals at day 21 post-challenge. Moreover, our results showed that Tp0663 and Tp0136 achieved high levels of serum antibodies against each protein after immunization. These antibodies possibly facilitated the retention of *T. pallidum* at the primary lesion sites by two potential mechanisms. One mechanism could be that the antibodies inhibited treponemal dissemination *via* blocking Tp0136-mediated invasion, which would be consistent with a previous study that showed Tp0136-specific serum inhibited the attachment of *T. pallidum* to both plasma and cellular Fn (13). The other mechanism could be that the antibodies opsonized *T. pallidum*, which promoted its clearance through opsonophagocytosis since Tp0663 and Tp0136 are both surface-exposed outer membrane proteins (10, 14). It is likely that both of these mechanisms may contribute to the function of Tp0663 and Tp0136 in protecting against *T. pallidum* dissemination. However, the specific mechanism remains to be further studied. Furthermore, the Tp0663-immunized or Tp0136-immunized animals showed a higher level of Th1 cytokine IFN-γ when compared to control animals, which was in accordance with the fact that the IFN-γ-dominated Th1 response facilitates the elimination of *T. pallidum* in the early infection stage (31).

Tp0136 was previously hypothesized to be a syphilis vaccine candidate (10). Since Tp0136 reacts strongly with serum antibodies from *T. pallidum-*infected rabbits and syphilis patients and can bind to host Fn and laminin (13), we preceded with rabbit vaccination experiments. Our findings were in agreement with the previous phenomenon that a significant delay in ulceration was observed in Tp0136-immunized rabbits (10). In addition, we further found that the recombinant Tp0136 protein was able to protect against *T. pallidum* dissemination from the primary site of infection to distant organs, indicating that Tp0136 may be a promising syphilis vaccine candidate. Owing to the fact that Tp0136 gene is extremely variable, it is possible that further work with the regions that are completely conserved among strains may generate better immune protection. Moreover, it is a good strategy to formulate a multi-component vaccine consisting of Tp0136 and other protective antigens. Although the surface-exposed outer membrane proteins are thought to be the most promising syphilis vaccine candidates at present, the identification of outer membrane proteins are elusive (22, 43). Brinkman et al. first showed that Tp0136 was surface localized (10), but then Cox et al. found that Tp0136 was periplasmic (44). Thus, it is necessary to determine the position of Tp0136 in *T. pallidum*, which may contribute to clarifying the protective mechanism of the recombinant Tp0136 protein against *T. pallidum*.

Although Tp0663 has not been well studied, some researchers have suggested that it has surface-exposed epitopes and could be reactive with sera from syphilis patients and New Zealand rabbits infected with the *T. pallidum* (14–16), suggesting that Tp0663 is readily expressed by *T. pallidum*. Although several individual antigens have previously been selected for immunization and tested for their protective capacity in rabbit models, only a few outer membrane proteins were proven to prevent chancre development. In this study, rabbits immunized with Tp0663 showed significantly delayed chancre development. In addition, unlike other outer membrane proteins, the chancre presented in Tp0663-immunized rabbits healed quickly, even compared to Tp0136-immunized rabbits and unimmunized rabbits. Similarly, qPCR and dark-field microscopy analysis suggested that the treponemal burden at the primary lesion sites of Tp0663-immunized rabbits were far less than that found in the primary lesion sites of Tp0136-immunized and unimmunized rabbits. This is probably because the anti-Tp0663 antibodies may have aided the macrophages in removing the majority of *T. pallidum*. However, the concrete mechanism needs further study.

Although this study exhibited promising results regarding the efficacy of Tp0136 and Tp0663 as syphilis vaccine candidates, it should not be ignored that the statistical power was limited because of the very small sample size of 3 rabbits per group as well as the fact that the rabbits were outbred. However, this pilot study can provide parameters for future investigations. Another limitation of this study was the use of an extremely high challenge dose. Challenge with a lower dose would likely have revealed more significant differences in lesion development. In addition, non-living vaccine antigens, such as single recombinant antigens, are often poorly immunogenic and require an adjuvant to help stimulate protective immunity based on antibodies and effector T cell functions. Although complete Freund’s adjuvant is unacceptable for human use due to undesirable side effects attributed to its use (45), Freund’s adjuvant is effective at activating innate immunity and developing antigen-specific T and B lymphocytes (46). Moreover, complete Freund’s adjuvant is also effective in stimulating a cellular immune response and may potentiate the production of IgG and IgA (47). Complete Freund’s adjuvant was used to enhance the immune response and preliminarily assess the vaccine potential of Tp0136 and Tp0663 in this study. However, clinically used and tested adjuvants should be used in further experiments.

In summary, the results presented in our study indicate that the *T. pallidum* adhesion protein Tp0136 and the outer membrane protein Tp0663 are promising vaccine candidates. Immunization with these proteins significantly attenuated lesion development and reduced treponemal dissemination within the host, resulting in protection similar to that with Tp0751 (28). Preventing the dissemination of *T. pallidum* is a vital requirement for a syphilis vaccine, as it would prevent the development of all stages of syphilis and the transmission of congenital syphilis. These results provide novel and important information for the understanding *T. pallidum* pathogenesis. Furthermore, these studies also provide insights into the development of spirochetal subunit vaccines, which may offer a timely solution to confront public health-targeted syphilis elimination.

## Data Availability Statement

The original contributions presented in the study are included in the article/supplementary material. Further inquiries can be directed to the corresponding author.

## Ethics Statement

All of the animal studies were approved by the Ethics Committee of the University of South China.

## Author Contributions

The experiment was designed by YX, MX, and YW, and completed by MX, HL, and KZ. The statistics of the experimental data were done by MT, FZ, and TZ together. All authors contributed to this article and approved the submitted version.

## Funding

This work was supported by the National Natural Science Foundation of China (grant numbers 82002182, 81702046, and 81471576), Natural Science Foundation of Hunan Province (grant numbers 2021JJ40479 and 2019JJ50535), Hunan Province Cooperative Innovation Center for Molecular Target New Drug Study (grant number 2015-351), and Hunan Provincial Key Laboratory for Special Pathogens Prevention and Control Foundation (grant number 2014-5).

## Conflict of Interest

The authors declare that the research was conducted in the absence of any commercial or financial relationships that could be construed as a potential conflict of interest.

## Publisher’s Note

All claims expressed in this article are solely those of the authors and do not necessarily represent those of their affiliated organizations, or those of the publisher, the editors and the reviewers. Any product that may be evaluated in this article, or claim that may be made by its manufacturer, is not guaranteed or endorsed by the publisher.

## References

[1] Newman L Rowley J Vander Hoorn S Wijesooriya NS Unemo M Low N. Global Estimates of the Prevalence and Incidence of Four Curable Sexually Transmitted Infections in 2012 Based on Systematic Review and Global Reporting. PloS One (2015) 10:e0143304. doi: 10.1371/journal.pone.0143304 26646541 PMC4672879

[2] De Voux A Kidd S Grey JA Rosenberg ES Gift TL Weinstock H. State-Specific Rates of Primary and Secondary Syphilis Among Men Who Have Sex With Men - United States, 2015. MMWR Morb Mortal Wkly Rep (2017) 66:349–54. doi: 10.15585/mmwr.mm6613a1 PMC5657910 28384130

[3] Jansen K Schmidt AJ Drewes J Bremer V Marcus U. Increased Incidence of Syphilis in Men Who Have Sex With Men and Risk Management Strategies, Germany, 2015. Euro Surveill (2016) 21:30382. doi: 10.2807/1560-7917.ES.2016.21.43.30382 PMC5114722 27813472

[4] Botham SJ Ressler KA Maywood P Hope KG Bourne CP Conaty SJ. Men Who Have Sex With Men, Infectious Syphilis and HIV Coinfection in Inner Sydney: Results of Enhanced Surveillance. Sex Health (2013) 10:291–8. doi: 10.1071/SH12142 23639847

[5] Burchell AN Allen VG Gardner SL Moravan V Tan DH Grewal R. High Incidence of Diagnosis With Syphilis Co-Infection Among Men Who Have Sex With Men in an HIV Cohort in Ontario, Canada. BMC Infect Dis (2015) 15:356. doi: 10.1186/s12879-015-1098-2 26289937 PMC4546079

[6] Petersen J Gibin M Sile B Simms I. Identifying and Interpreting Spatiotemporal Variation in Diagnoses of Infectious Syphilis Among Men, England: 2009 to 2013. Sex Transm Infect (2016) 92:380–6. doi: 10.1136/sextrans-2015-052306 27147614

[7] Chen G Cao Y Yao Y Li M Tang W Li J. Syphilis Incidence Among Men Who Have Sex With Men in China: Results From a Meta-Analysis. Int J STD AIDS (2017) 28:170–8. doi: 10.1177/0956462416638224 PMC5026914 26992411

[8] Newman L Kamb M Hawkes S Gomez G Say L Seuc A. Global Estimates of Syphilis in Pregnancy and Associated Adverse Outcomes: Analysis of Multinational Antenatal Surveillance Data. PloS Med (2013) 10:e1001396. doi: 10.1371/journal.pmed.1001396 23468598 PMC3582608

[9] Kidd S Torrone E Su J Weinstock H. Reported Primary and Secondary Syphilis Cases in the United States: Implications for HIV Infection. Sex Transm Dis (2018) 45:S42–7. doi: 10.1097/OLQ.0000000000000810 PMC6745698 29465633

[10] Brinkman MB Mcgill MA Pettersson J Rogers A Matejkova P Smajs D. A Novel Treponema Pallidum Antigen, TP0136, Is an Outer Membrane Protein That Binds Human Fibronectin. Infect Immun (2008) 76:1848–57. doi: 10.1128/IAI.01424-07 PMC2346692 18332212

[11] Luo X Gao ZX Lin SW Tong ML Liu LL Lin LR. Recombinant Treponema Pallidum Protein Tp0136 Promotes Fibroblast Migration by Modulating MCP-1/CCR2 Through TLR4. J Eur Acad Dermatol Venereol (2020) 34:862–72. doi: 10.1111/jdv.16162 31856347

[12] Luo X Lin SW Xu QY Ke WJ Gao ZX Tong ML. Tp0136 Targets Fibronectin (RGD)/Integrin Beta1 Interactions Promoting Human Microvascular Endothelial Cell Migration. Exp Cell Res (2020) 396:112289. doi: 10.1016/j.yexcr.2020.112289 32950474

[13] Ke W Molini BJ Lukehart SA Giacani L. Treponema Pallidum Subsp. Pallidum TP0136 Protein Is Heterogeneous Among Isolates and Binds Cellular and Plasma Fibronectin *via* Its NH2-Terminal End. PloS Negl Trop Dis (2015) 9:e0003662. doi: 10.1371/journal.pntd.0003662 25793702 PMC4368718

[14] Champion CI Blanco DR Exner MM Erdjument-Bromage H Hancock RE Tempst P. Sequence Analysis and Recombinant Expression of a 28-Kilodalton Treponema Pallidum Subsp. Pallidum Rare Outer Membrane Protein (Tromp2). J Bacteriol (1997) 179:1230–8. doi: 10.1128/jb.179.4.1230-1238.1997 PMC178820 9023206

[15] Mckevitt M Brinkman MB Mcloughlin M Perez C Howell JK Weinstock GM. Genome Scale Identification of Treponema Pallidum Antigens. Infect Immun (2005) 73:4445–50. doi: 10.1128/IAI.73.7.4445-4450.2005 PMC1168556 15972547

[16] Brinkman MB Mckevitt M Mcloughlin M Perez C Howell J Weinstock GM. Reactivity of Antibodies From Syphilis Patients to a Protein Array Representing the Treponema Pallidum Proteome. J Clin Microbiol (2006) 44:888–91. doi: 10.1128/JCM.44.3.888-891.2006 PMC1393150 16517872

[17] Lithgow KV Cameron CE. Vaccine Development for Syphilis. Expert Rev Vaccines (2017) 16:37–44. doi: 10.1080/14760584.2016.1203262 27328030 PMC5513191

[18] Cullen PA Cameron CE. Progress Towards an Effective Syphilis Vaccine: The Past, Present and Future. Expert Rev Vaccines (2006) 5:67–80. doi: 10.1586/14760584.5.1.67 16451109

[19] Miller JN. Immunity in Experimental Syphilis. VI. Successful Vaccination of Rabbits With Treponema Pallidum, Nichols Strain, Attenuated by -Irradiation. J Immunol (1973) 110:1206–15. 4572631

[20] Giacani L Sambri V Marangoni A Cavrini F Storni E Donati M. Immunological Evaluation and Cellular Location Analysis of the Tpri Antigen of Treponema Pallidum Subsp. Pallidum. Infect Immun (2005) 73:3817–22. doi: 10.1128/IAI.73.6.3817-3822.2005 PMC1111852 15908421

[21] Morgan CA Lukehart SA Van Voorhis WC. Immunization With the N-Terminal Portion of Treponema Pallidum Repeat Protein K Attenuates Syphilitic Lesion Development in the Rabbit Model. Infect Immun (2002) 70:6811–6. doi: 10.1128/IAI.70.12.6811-6816.2002 PMC133068 12438357

[22] Cameron CE Lukehart SA Castro C Molini B Godornes C Van Voorhis WC. Opsonic Potential, Protective Capacity, and Sequence Conservation of the Treponema Pallidum Subspecies Pallidum Tp92. J Infect Dis (2000) 181:1401–13. doi: 10.1086/315399 10762571

[23] Borenstein LA Radolf JD Fehniger TE Blanco DR Miller JN Lovett MA. Immunization of Rabbits With Recombinant Treponema Pallidum Surface Antigen 4D Alters the Course of Experimental Syphilis. J Immunol (1988) 140:2415–21. 2450921

[24] Cameron CE Castro C Lukehart SA Van Voorhis WC. Function and Protective Capacity of Treponema Pallidum Subsp. Pallidum Glycerophosphodiester Phosphodiesterase. Infect Immun (1998) 66:5763–70. doi: 10.1128/IAI.66.12.5763-5770.1998 PMC108728 9826352

[25] Sun ES Molini BJ Barrett LK Centurion-Lara A Lukehart SA Van Voorhis WC. Subfamily I Treponema Pallidum Repeat Protein Family: Sequence Variation and Immunity. Microbes Infect (2004) 6:725–37. doi: 10.1016/j.micinf.2004.04.001 15207819

[26] Champion CI Miller JN Borenstein LA Lovett MA Blanco DR. Immunization With Treponema Pallidum Endoflagella Alters the Course of Experimental Rabbit Syphilis. Infect Immun (1990) 58:3158–61. doi: 10.1128/iai.58.9.3158-3161.1990 PMC313628 2201648

[27] Zheng K Xu M Xiao Y Luo H Xie Y Yu J. Immunogenicity and Protective Efficacy Against Treponema Pallidum in New Zealand Rabbits Immunized With Plasmid DNA Encoding Flagellin. Emerg Microbes Infect (2018) 7:177. doi: 10.1038/s41426-018-0176-0 30405111 PMC6220273

[28] Lithgow KV Hof R Wetherell C Phillips D Houston S Cameron CE. A Defined Syphilis Vaccine Candidate Inhibits Dissemination of Treponema Pallidum Subspecies Pallidum. Nat Commun (2017) 8:14273. doi: 10.1038/ncomms14273 28145405 PMC5296639

[29] Lukehart SA Marra CM. Isolation and Laboratory Maintenance of Treponema Pallidum. Curr Protoc Microbiol (2007)Chapter 12:Unit 12A 11. doi: 10.1002/9780471729259.mc12a01s7 18770607

[30] Xu M Xie Y Jiang C Xiao Y Kuang X Zhao F. A Novel ELISA Using a Recombinant Outer Membrane Protein, Rtp0663, as the Antigen for Serological Diagnosis of Syphilis. Int J Infect Dis (2016) 43:51–7. doi: 10.1016/j.ijid.2015.12.013 26747418

[31] Leader BT Godornes C Vanvoorhis WC Lukehart SA. CD4+ Lymphocytes and Gamma Interferon Predominate in Local Immune Responses in Early Experimental Syphilis. Infect Immun (2007) 75:3021–6. doi: 10.1128/IAI.01973-06 PMC1932874 17403876

[32] Lafond RE Lukehart SA. Biological Basis for Syphilis. Clin Microbiol Rev (2006) 19:29–49. doi: 10.1128/CMR.19.1.29-49.2006 16418521 PMC1360276

[33] Salazar JC Rathi A Michael NL Radolf JD Jagodzinski LL. Assessment of the Kinetics of Treponema Pallidum Dissemination Into Blood and Tissues in Experimental Syphilis by Real-Time Quantitative PCR. Infect Immun (2007) 75:2954–8. doi: 10.1128/IAI.00090-07 PMC1932886 17438037

[34] Baker-Zander SA Lukehart SA. Macrophage-Mediated Killing of Opsonized Treponema Pallidum. J Infect Dis (1992) 165:69–74. doi: 10.1093/infdis/165.1.69 1727898

[35] Baker-Zander SA Shaffer JM Lukehart SA. Characterization of the Serum Requirement for Macrophage-Mediated Killing of Treponema Pallidum Ssp. Pallidum: Relationship to the Development of Opsonizing Antibodies. FEMS Immunol Med Microbiol (1993) 6:273–9. doi: 10.1111/j.1574-695X.1993.tb00339.x 8499892

[36] Shaffer JM Baker-Zander SA Lukehart SA. Opsonization of Treponema Pallidum Is Mediated by Immunoglobulin G Antibodies Induced Only by Pathogenic Treponemes. Infect Immun (1993) 61:781–4. doi: 10.1128/iai.61.2.781-784.1993 PMC302795 8423106

[37] Edmondson DG Hu B Norris SJ. Long-Term *In Vitro* Culture of the Syphilis Spirochete Treponema Pallidum Subsp. Pallidum. MBio (2018) 9:e01153-18. doi: 10.1128/mBio.01153-18 29946052 PMC6020297

[38] Van Voorhis WC Barrett LK Nasio JM Plummer FA Lukehart SA. Lesions of Primary and Secondary Syphilis Contain Activated Cytolytic T Cells. Infect Immun (1996) 64:1048–50. doi: 10.1128/iai.64.3.1048-1050.1996 PMC173879 8641758

[39] Cruz AR Ramirez LG Zuluaga AV Pillay A Abreu C Valencia CA. Immune Evasion and Recognition of the Syphilis Spirochete in Blood and Skin of Secondary Syphilis Patients: Two Immunologically Distinct Compartments. PloS Negl Trop Dis (2012) 6:e1717. doi: 10.1371/journal.pntd.0001717 22816000 PMC3398964

[40] Carlson JA Dabiri G Cribier B Sell S. The Immunopathobiology of Syphilis: The Manifestations and Course of Syphilis Are Determined by the Level of Delayed-Type Hypersensitivity. Am J Dermatopathol (2011) 33:433–60. doi: 10.1097/DAD.0b013e3181e8b587 PMC3690623 21694502

[41] Moore MW Cruz AR Lavake CJ Marzo AL Eggers CH Salazar JC. Phagocytosis of Borrelia Burgdorferi and Treponema Pallidum Potentiates Innate Immune Activation and Induces Gamma Interferon Production. Infect Immun (2007) 75:2046–62. doi: 10.1128/IAI.01666-06 PMC1865718 17220323

[42] Hawley KL Cruz AR Benjamin SJ La Vake CJ Cervantes JL Ledoyt M. Ifngamma Enhances CD64-Potentiated Phagocytosis of Treponema Pallidum Opsonized With Human Syphilitic Serum by Human Macrophages. Front Immunol (2017) 8:1227. doi: 10.3389/fimmu.2017.01227 29051759 PMC5633599

[43] Radolf JD. Treponema Pallidum and the Quest for Outer Membrane Proteins. Mol Microbiol (1995) 16:1067–73. doi: 10.1111/j.1365-2958.1995.tb02332.x 8577243

[44] Cox DL Luthra A Dunham-Ems S Desrosiers DC Salazar JC Caimano MJ. Surface Immunolabeling and Consensus Computational Framework to Identify Candidate Rare Outer Membrane Proteins of Treponema Pallidum. Infect Immun (2010) 78:5178–94. doi: 10.1128/IAI.00834-10 PMC2981305 20876295

[45] Sivakumar SM Safhi MM Kannadasan M Sukumaran N. Vaccine Adjuvants - Current Status and Prospects on Controlled Release Adjuvancity. Saudi Pharm J (2011) 19:197–206. doi: 10.1016/j.jsps.2011.06.003 23960760 PMC3744968

[46] Coffman RL Sher A Seder RA. Vaccine Adjuvants: Putting Innate Immunity to Work. Immunity (2010) 33:492–503. doi: 10.1016/j.immuni.2010.10.002 21029960 PMC3420356

[47] Billiau A Matthys P. Modes of Action of Freund’s Adjuvants in Experimental Models of Autoimmune Diseases. J Leukoc Biol (2001) 70:849–60. 11739546

